# Risk for COVID-19 Vulnerability in Patients with Inflammatory Bowel Disease: Assessing Alterations in ACE2 and TMPRSS2

**DOI:** 10.3390/biomedicines13092240

**Published:** 2025-09-11

**Authors:** Jorge Sáez-Leyva, Matthew P. Lennol, Carlos Avilés-Granados, María-Salud García-Ayllón, Javier Sáez-Valero

**Affiliations:** 1 Instituto de Neurociencias de Alicante, Universidad Miguel Hernández-CSIC, 03550 San Juan de Alicante, Spain; jorge.saez01@goumh.umh.es (J.S.-L.); caviles@umh.es (C.A.-G.); 2 Centro de Investigación Biomédica en Red sobre Enfermedades Neurodegenerativas (CIBERNED), 03550 San Juan de Alicante, Spain; 3 Institute of Neuro Physiopathology (INP), CNRS UMR 7051, Aix-Marseille University, 13005 Marseille, France; matthew.LENNOL@univ-amu.fr; 4 Instituto de Investigación Sanitaria y Biomédica de Alicante (ISABIAL), 03010 Alicante, Spain; 5 Unidad de Investigación, Hospital General Universitario de Elche, FISABIO, 03203 Elche, Spain

**Keywords:** ACE2, ADAM17, Crohn’s disease, inflammatory bowel disease, plasma, therapy, ulcerative colitis, TMPRSS2

## Abstract

Chronic inflammatory conditions often involve the dysregulation of key enzymes, including serine proteases such as transmembrane serine protease 2 (TMPRSS2) and the angiotensin converting enzyme 2 (ACE2), which are key proteins implicated in the cellular entry mechanism of SARS-CoV-2. It remains uncertain whether the gastrointestinal symptoms observed in COVID-19 patients result from direct viral infection of the gastrointestinal tract, a process that may be exacerbated by altered expression of ACE2 or TMPRSS2. In this review, we explore the interplay among ACE2 and TMPRSS2 in the context of inflammatory bowel disease (IBD), including their roles in disease pathology and response to therapy. We also examine methodological approaches for assessing whether protease alterations contribute to increased susceptibility to infection, considering that TMPRSS2 exists in inactive (zymogen) and active forms. Furthermore, while membrane-bound ACE2 facilitates viral entry, soluble ACE2 fragments may act as decoys, preventing virus–receptor interaction. Therefore, the interpretation of changes in full-length versus cleaved forms of ACE2 and related enzymes is critical for understanding vulnerability to SARS-CoV-2 infection.

## 1. Introduction

### 1.1. Inflammatory Bowel Disease (IBD)

At the turn of the 21st century, IBD emerged as a growing global health concern, exerting an increasing burden on healthcare systems worldwide. The prevalence of IBD now exceeds 0.3% in many regions [1], with incidence rates continuing to rise, particularly in newly industrialized countries experiencing rapid urbanization and westernization. Projections suggest that the prevalence may surpass 1% of the population in industrialized nations within the next decade [2], underscoring the urgent need for enhanced strategies in prevention, diagnosis, and long-term disease management.

**Figure 1:**
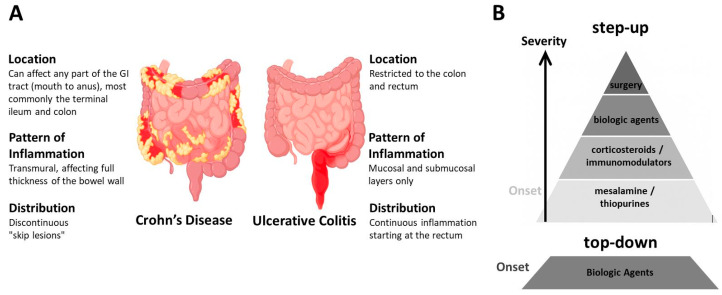
Crohn’s disease and ulcerative colitis: distinguishing features and common treatment. (**A**) Schematic representation illustrating the key differences between the two main forms of inflammatory bowel disease (IBD): Crohn’s disease (CD) and ulcerative colitis (UC), in terms of their typical location, inflammation pattern, and distribution (highlighted in red) along the gastrointestinal tract. (**B**) IBD treatment is typically tailored based on disease severity, prior treatment response, and individual patient factors, following either a “step-up” or “top-down” therapeutic approach. Surgical intervention is considered in cases of complications such as strictures, perforation, failure of medical therapy, or increased cancer risk.

Crohn’s disease (CD) and ulcerative colitis (UC) are the two principal subtypes of inflammatory bowel disease (IBD). The most prevalent clinical symptoms of IBD include chronic bloody diarrhea, abdominal pain, rectal bleeding, weight loss, and fatigue. Systemic features such as fever and malaise are also frequently reported, particularly during disease flares. Although UC and CD share overlapping clinical manifestations, these conditions differ markedly in their pathophysiology, anatomical distribution, and histopathological features. UC is characterized by a continuous mucosal inflammation that typically originates in the rectum and extends proximally through the colon, without affecting other regions of the gastrointestinal tract. Histologically, UC is associated with a reduced number of intestinal crypts and mucosal disruption, accompanied by significant infiltration of immune cells, including macrophages, lymphocytes, granulocytes, and plasma cells. In contrast, CD is defined by a transmural, segmental, and asymmetrical pattern of inflammation that can affect any part of the gastrointestinal tract, with the terminal ileum and colon being the most commonly involved sites. Histopathological examination of CD-affected tissue reveals disrupted intestinal crypts, transmural inflammatory infiltrates, and the presence of lymphoid aggregates (reviewed in [3,4]) (Figure 1A).

Currently, all therapeutic interventions for IBD are directed at controlling inflammation; however, they are ineffective in reversing established chronic bowel wall damage. Treatment decisions are primarily based on symptom severity and traditionally follow a step-up approach [5], but when treatment is personalized, it may shift to a top-down approach depending on the disease course and patient needs [6,7] (Figure 1B). The goal of treatment is to induce and maintain remission, prevent complications, and improve quality of life [8]. Accordingly, initial therapy for mild presentations of the disease often includes mesalamine/5-aminosalicylic acid [9] and immunomodulators such as thiopurines (e.g., azathioprine) [10]. Azathioprine is the immunomodulator of choice for moderately active IBD and is intended to reduce inflammation; however, its efficacy in maintaining remission is limited [11], particularly when used as monotherapy [12]. Corticosteroids are potent anti-inflammatory and immunosuppressive agents widely used to manage both acute and chronic inflammatory conditions, including moderate-to-severe IBD. Nevertheless, their use is constrained by a range of significant adverse effects [13].

When more potent therapies are required, treatment typically progresses to biologic agents, most commonly monoclonal antibodies targeting tumor necrosis factor-α (TNFα), such as infliximab and adalimumab, or targeting specific interleukins (IL-12/23), such as ustekinumab, or targeting integrins such as vedolizumab [14]. As previously noted, in the top-down therapeutic approach, biologics may be introduced early in the disease course to prevent progression and complications. The advent of biologic therapies has significantly transformed IBD management, leading to improved early treatment responses and unprecedented outcomes, including complete mucosal and histologic healing, consequently reducing hospitalization rates. Additionally, Janus kinase (JAK) inhibitors represent a novel class of therapeutic agents that modulate dysregulated immune responses by attenuating multiple cytokine signaling pathways implicated in the pathogenesis of IBD [15]. Tofacitinib, filgotinib, and upadacitinib have been approved for the treatment of moderate-to-severe UC. Among them, upadacitinib is currently the only JAK inhibitor also approved for the treatment of CD [15]. Application of opioids, probiotics or fecal microbiota is also explored in the treatment of IBD [16].

### 1.2. IBD and COVID-19

The spread of COVID-19 to countries with high incidence rates of IBD, along with evidence that SARS-CoV-2 can infect gastrointestinal cells (reviewed in [17]), has raised early concerns about the severity of COVID-19 in IBD patients. Although the underlying mechanisms connecting IBD and COVID-19 remain poorly understood, it has been proposed that IBD patients could be more vulnerable to COVID-19 [18]. Moreover, a large number of COVID-19 cases experience digestive symptoms [19,20], whereas gastrointestinal symptoms are part of the manifestations in long COVID-19 [21], and a case report even suggested that COVID-19 could trigger UC [22].

Anyhow, several studies have indicated that the prevalence of COVID-19 in patients with IBD was similar to the general population [23] and even to household members [24]; patients with other immune-mediated inflammatory diseases also showed lower susceptibility to COVID-19 [25]. Moreover, individuals with IBD do not have an increased risk of COVID-19 associated mortality, or with intensive care unit admission compared to the general population [26,27]. Nonetheless, most of the available evidence regarding the impact of COVID-19 in IBD derives from observational studies (reviewed in [28]), and few studies have investigated whether underlying pathobiological features in IBD patients confer increased susceptibility to local gastrointestinal SARS-CoV-2 infection.

For that reason, the potential contribution of chronic gastrointestinal inflammation, or altered expression of key proteins involved in SARS-CoV-2 cell entry, to the increase in local infection rates should not be overlooked. Indeed, chronic inflammatory conditions have been proposed as potential risk factors for increased COVID-19 severity [29], and an excessive inflammatory state increases the risk of severe disease and mortality in patients with COVID-19 [30]. Previous reviews addressed SARS-CoV-2 effects on the gastrointestinal systems, including the inflammatory response [31], however few studies investigated whether key proteins involved in SARS-CoV-2 infection are altered in association with IBD pathology. An angiotensin-converting enzyme 2 (ACE2) upregulation in active IBD tissue has been linked to compensatory anti-inflammatory responses (discussed in [32]) but may also result in increased SARS-CoV-2 vulnerability in people with gastrointestinal infections.

In this review, we examine whether the expression of key host proteins required for viral entry, namely ACE2, which serves as the cellular receptor for SARS-CoV-2, and the transmembrane serine protease 2 (TMPRSS2), which primes the viral spike protein, are altered in individuals with IBD. We discussed the implications of alterations in UC and in CD for particular molecular forms of ACE2 and TMPRSS2 in relation to gastrointestinal susceptibility to SARS-CoV-2 infection. Accordingly, we examine the accurate interpretation of changes in tissue biopsies, as well as in circulating molecular forms, where co-existing shedded fragments of ACE2 and TMPRSS2 are also present, alongside full-length species.

## 2. Characterization of ACE2 and TMPRSS2 Molecular Forms and Significance for COVID-19 Risk

As mentioned above, there is consensus regarding SARS-CoV entry into host cells via the gastrointestinal tract by binding to the enterocyte-expressed ACE2 receptor; and its colocalization with TMPRSS2 on cell surfaces, which facilitates viral spike fusogenic activity and also promotes viral entry [33]. ACE2 is predominantly located in human gastrointestinal-tract epithelia, and the highest levels of TMPRSS2 are also found in the gastrointestinal tract [34]. In particular, ACE2 is expressed largely in the colon and the terminal ileum [35].

### 2.1. ACE2 Protein Function and Proteolytic Processing

ACE2 is a monocarboxypeptidase that serves as a key counter-regulatory component of the renin-angiotensin system (RAS). The RAS is one of the most complex hormonal regulatory systems, involving several organs that interact to regulate multiple body functions [36]. The canonical role of RAS in the regulation of cardiovascular function has been extended by evidence that local RAS exists in diverse tissue types and by findings that indicate the RAS comprises two axes, the classic RAS and an alternative RAS, with antagonistic effects that are usually in equilibrium. The classic system is involved in pathologies where inflammatory, hypertrophic and fibrotic phenomena are common and are related to the development of chronic diseases that affect various body systems. In the classical RAS pathway, renin converts angiotensinogen into angiotensin I (Ang I), which is subsequently cleaved by angiotensin-converting enzyme (ACE) to form angiotensin II (Ang II). Ang II, through activation of the angiotensin II type 1 receptor (AT1R), exerts potent vasoconstrictive, pro-inflammatory, pro-thrombotic, and pro-fibrotic effects. In the alternative axis, ACE2 mitigates the effects of this axis by degrading Ang II into angiotensin-(1–7) [Ang-(1–7)], which binds to the Mas receptor and initiates signaling cascades that promote anti-fibrotic, anti-proliferative and anti-inflammatory effects [37]. By reducing Ang II bioavailability and simultaneously increasing Ang-(1–7) levels, ACE2 effectively shifts the RAS balance toward a protective balance within the system. Thus, enzymatic activity of ACE2 is essential for cardiovascular and renal physiology, as it regulates blood pressure, vascular tone, sodium and water balance, and prevents maladaptive tissue remodeling. Dysregulation of ACE2 expression or activity has been implicated in hypertension, heart failure, chronic kidney disease, acute lung injury, among others, and has potential implications in SARS-CoV vulnerability [38,39,40]. In contrast, Ang-(1–7) binds to the Mas receptor and initiates signaling cascades that promote vasodilation, anti-inflammatory responses, anti-proliferative effects, and tissue protection [41]. By reducing Ang II bioavailability and simultaneously increasing Ang-(1–7) levels, ACE2 effectively shifts the RAS balance toward a vasoprotective and homeostatic state. Moreover, ACE2 presents other physiological implications in RAS, since it can also drive cleavage of other vasoactive peptides such as the Mas receptor and AT2R [42]. ACE2 can also act as a partner for intestinal amino acid transporters [43]. For a complete review of ACE2, see [44].

The relationship between ACE2 and gastrointestinal disease will be discussed later, but components of the RAS, such as Ang-(1–7) and Mas receptor have been implicated in IBD [45], suggesting that an imbalance of the RAS may contribute to inflammation and fibrosis in IBD [46]. In this context, drugs targeting RAS have demonstrated anti-inflammatory and antifibrotic properties in the gut in preclinical studies, offering potential benefits for IBD patients (reviewed in [47]).

**Figure 2:**
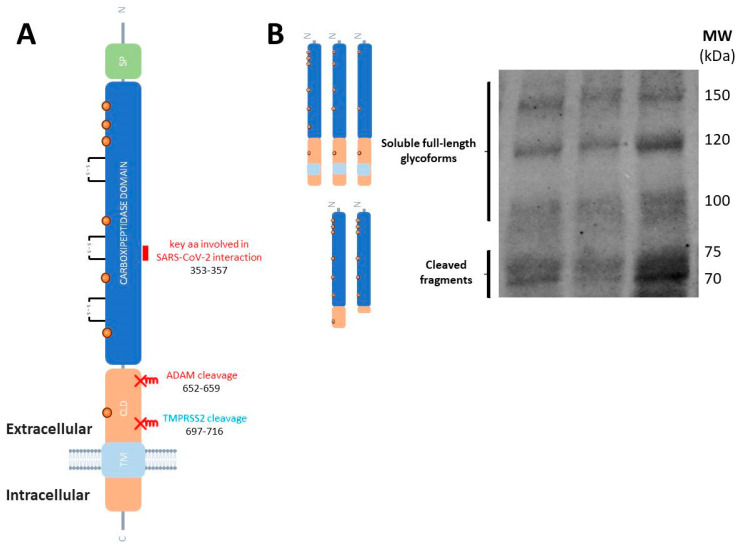
Molecular species of ACE2 in human tissues and fluids. (**A**) Schematic representation of ACE2 as a transmembrane type I glycoprotein (not drawn to scale). The single peptide (SD), carboxypeptidase domain, collectrin-like domain (CLD) and the transmembrane (TM) domain are represented. SARS-CoV-2 S-protein binds to the carboxypeptidase ectodomain. The short domain (key amino acid residues 353-KGDFR-357) involved in the interaction with SARS-CoV-2 spike glycoprotein is represented (marked in purple). The sites of ACE2 shedding with ADAM17 are also indicated. (**B**) Representative blot of human plasma samples incubated with the anti-ACE2 ectodomain AF933 antibody (R&D Systems) showing the cleaved ACE2 fragments, and the soluble full-length species which diversity could be attributed to highly glycosylated forms (unpublished blot).

Some of these biological functions of ACE2 are exerted by soluble species. These soluble forms are generated by the cleavage of the membrane-resident ACE2 by the membrane-bound sheddases from the ADAM (a disintegrin and metalloprotease) family, ADAM17/TACE and ADAM10 [48], generating large ectodomain fragments that retain the carboxypeptidase [49] and the binding region to SARS-CoV-2 [50] (Figure 2A). It has been proposed that TMPRSS2 and ADAM17 will compete for ACE2 cleavage, resulting in different ACE2 cleaved products, which will exhibit differential fates [51]. ACE2 cleavage by ADAM17 and ADAM10 occurs through constitutive and regulated shedding [49], while it is unclear whether the cleavage of ACE2 by TMPRSS2 occurs in the absence of the coronavirus.

Interestingly, it has been demonstrated that full-length, unprocessed ACE2 retaining both its transmembrane and cytoplasmic domains is present as soluble forms in human plasma (Figure 2B), cerebrospinal fluid (CSF), and other biological fluids [52,53]. As a result, both cleaved ACE2 fragments and full-length ACE2 species may contribute in different proportions to the total soluble pool of ACE2, with variations in pathological conditions. Indeed, the presence of ACE2 fragments in circulation is expected to result from the shedding of membrane-bound ACE2, and a previous study suggested that circulating levels of full-length ACE2 may reflect its tissue expression levels [54]. Nonetheless, the potential contribution of full-length ACE2 to total plasma ACE2 levels has not been adequately considered in most studies. Notably, many of these investigations have relied on enzyme-linked immunosorbent assays (ELISA), or immunohistological analysis in intestinal biopsies, which often do not distinguish between ACE2 fragments and the full-length protein, or discriminate adequately between different fragments, thus leaving uncertainty regarding the specific species present in circulation and their contributions.

### 2.2. TMPRSS2 Protein Function and Proteolytic Processing

**Figure 3:**
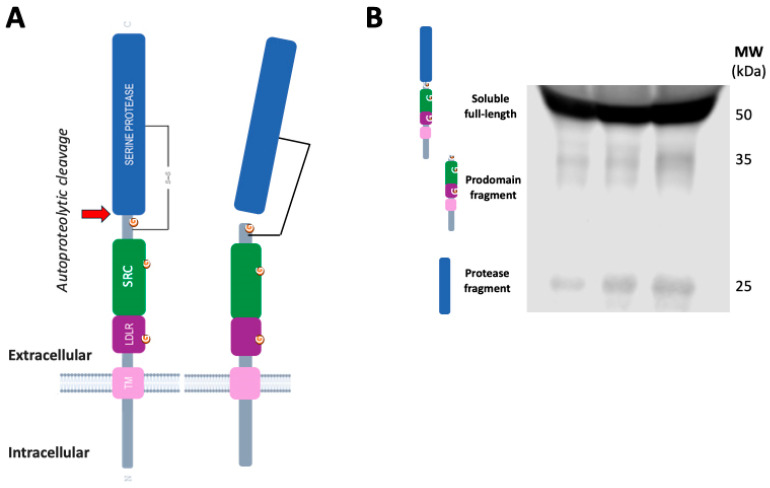
Molecular species of TMPRSS2 in human tissues and fluids. (**A**) Schematic representation of TMPRSS2 as a transmembrane type II protein (not drawn to scale). The serine protease domain, the scavenger receptor cysteine-rich (SRC) domain and the LDL-receptor class A domain (LDLR). TMPRSS2 is expressed as a single chain zymogen that undergoes autoproteolytic cleavage at the position indicated (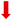, residues 255–256) to acquire proteolytic activity. The cleaved protease domain either remains linked to the prodomain via an interdomain disulfide bond or is shed, resulting in a membrane-bound fragment or a cell-free fragment. (**B**) Representative blot of human plasma samples incubated with an anti-TMPRSS2 ectodomain 14437-1-AP antibody (Proteintech) showing the TMPRSS2 soluble full-length form and both fragments, containing the peptidase domain and the N-terminal prodomain (unpublished blot).

Moreover, it has been demonstrated that infection of SARS-CoV-2 in mature enterocytes expressing ACE2 is facilitated by TMPRSS2, highlighting the intestine as a potential site of SARS-CoV-2 replication [55]. TMPRSS2 is also a transmembrane glycoprotein, which is synthesized as an inactive zymogen that undergoes autoproteolytic cleavage to generate an active serine protease domain (represented in Figure 3A), which is responsible for priming the SARS-CoV-2 spike protein. Following cleavage, the active protease domain may either remain associated with the pro-domain through an interdomain disulfide bond or is released into the extracellular environment in a soluble fragment form [56,57].

As mentioned above, TMPRSS2 displays a broad expression in epithelial cells of the respiratory and gastrointestinal tract, the prostate, and other organs, whose physiological role remains largely elusive. Several potential substrates for TMPRSS2 have been proposed, including protease-activated receptor-2 (PAR-2), which mediates protective functions in the gastrointestinal and respiratory tract; however, TMPRSS2 also cleaves pro-kallikrein-2 (KLK2) into mature KLK2 and activates the single-chain precursor of hepatocyte growth factor (HGF) (reviewed in [58]).

In human cells, the TMPRSS2 full-length zymogen is the most abundant species [55], and, surprisingly, this proteolytic unprocessed species is also the most abundant in plasma [59] and CSF [53] (Figure 3B). Therefore, it is also important to conduct an analysis by Western blotting, or techniques that discriminate between species, to estimate the levels of the protease fragment that could represent the active membrane-resident forms involved in SARS-CoV-2 cellular infection.

### 2.3. Significance of Particular ACE2 and TMPRSS2 Molecular Species Regarding COVID-19 Vulnerability

**Figure 4:**
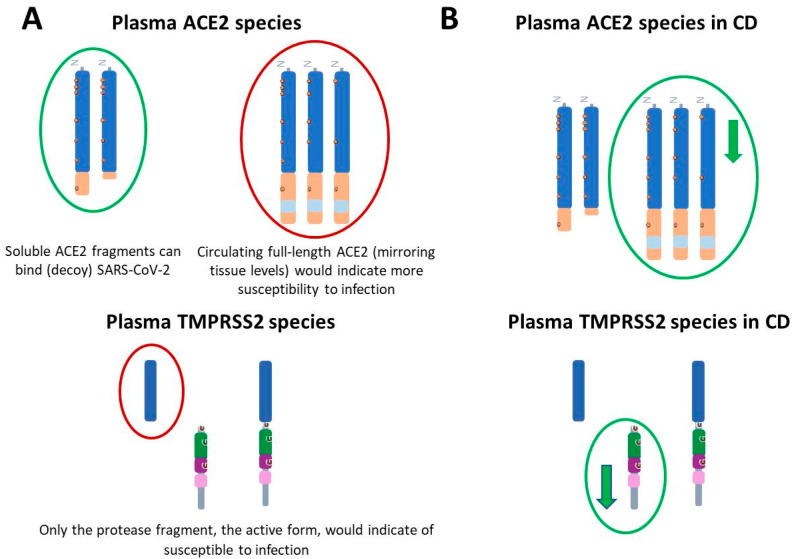
Significance of changes in molecular species of ACE2 and TMPRSS2 regarding COVID-19 and its alterations in CD. (**A**) Changes in ACE2 and TMPRSS2 associated with chronic conditions that might increase vulnerability to SARS-CoV-2 infection. Elevated levels of cleaved ACE2 fragments could be interpreted as potentially protective (circled in green), as soluble ACE2 may act as a decoy receptor by binding and neutralizing the virus. In this sense, increased active ADAM17 (a metalloprotease that cleaves ACE2) would also indicate less susceptibility to infection. Elevated levels of full-length species of ACE2, reflecting tissue content of the full-length membrane-resident forms or elevated levels of TMPRSS2 fragments could represent increased vulnerability (circled in red). (**B**) Levels of ACE2 and TMPRSS2 species in plasma from individuals affected by Crohn’s disease (CD). Full-length ACE2 decreased (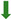) in the plasma of CD patients, as did the TMPRSS2 prodomain fragment, although both changes were unrelated with vulnerability to SARS-CoV2. For details see [59].

The importance of differentiating between tissue and circulating ACE2 regarding vulnerability to SARS-CoV-2 has been previously advised [60]. Vulnerability to SARS-CoV-2 infection should be assessed based on the relative abundance of specific circulating ACE2 species. Therefore, elevated levels of circulating ACE2 fragments could be interpreted as potentially protective, as soluble ACE2 may act as a decoy receptor by binding and neutralizing the virus [61]. On the other hand, high levels of membrane-resident ACE2 suggests increased vulnerability of the gastrointestinal tract to viral entry, and higher levels of membrane-resident ACE2 should also result in increased levels of circulating full-length ACE2 with protective effects [54]. With regard to TMPRSS2, increased vulnerability to SARS-CoV-2 should only be determined by elevated generation of active protease domains (Figure 4A includes a schematic representation).

## 3. Levels of ACE2 and TMPRSS2 in IBD Biopsy Tissue

To date, most studies investigating potential alterations in ACE2 and TMPRSS2 expression in IBD patients have relied on transcriptional analyses, yet no clear consensus has been established. ACE2 exhibits a heterogeneous expression pattern throughout the gastrointestinal tract [35], and both ACE2 and TMPRSS2 mRNA levels vary depending on the anatomical region and the presence of inflammation [62]. For that, although intestinal inflammation is a key factor influencing the expression of ACE2 and TMPRSS2 in IBD, regional differences within the gut also contribute to the observed variability [63]. Moreover, a particular ACE2 expression pattern could be related to the physiological roles of ACE2, including an alternative function that degrades Ang II. Hence, ACE2 presents other physiological implications in RAS, since it can also drive cleavage of other vasoactive peptides such as the Mas receptor and AT2R [42]. Beyond the RAS, ACE2 displays potential catalytic activity to cleave peptides like apelin-13, dynorphin 13, des-Arg9-bradykinin peptides, β-casomorphin, neurotensin 1–8 and ghrelin [64], and some of these substrates appear to be present in the gastrointestinal tract (reviewed in [65]).

ACE2 can also act as a partner for intestinal amino acid transporters [43], potentially playing other key physiological functions related to specific cellular and subcellular localizations and shedding, which could be affected across IBD pathology. In fact, it cannot be discarded that the cleaved intracellular domain of ACE2 might act as a transcriptional regulator analogous to the intracellular domain of amyloid precursor protein or the notch receptor following ectodomain cleavage (discussed in [44]).

The intracellular cholesterol transporter Niemann Pick C1 (NPC1) protein, located in the late endosome/lysosome, also influences ACE2 abundance at the plasma membrane and its inhibition relocalizes ACE2 from the plasma membrane to the autophagosomal/lysosomal compartment [66]. Moreover, although ACE2 colocalizes with raft-resident markers [67], incubation of cells with viral spike protein revealed discrete dotted patterns at the cell surface, which consistently colocalizes with endogenous ACE2, but sparsely with a lipid raft marker [68]. In fact, it has recently been reported that SARS-CoV-2 entry and fusion are independent of ACE2 localization to lipid rafts [69]. Thus, subcellular localization of ACE2, and TMPRSS2, should play a fundamental role in their biological function and involvement in pathology, but this issue warrants more investigation.

Moreover, variances among intracellular locations of ACE2 and TMPRSS2 could also contribute to a misinterpretation of changes in these proteins in the context of IBD and susceptibility to SARS-CoV-2 infectivity. In a recent study using immunohistochemistry, a significant increase in ACE2 and TMPRSS2 was reported in colon and rectal specimens of UC [70]; although in UC, colonic ACE2 and TMPRSS2 appear as cytoplasmic in nature [71], therefore these alterations could not determine changes in vulnerability to SARS-CoV-2, at least regarding cellular penetrance of the virus. A different study that reports downregulated transcript and protein levels of ACE2 in inflamed CD ileum, and in multiple well-established mouse models, also demonstrated altered localization of intestinal epithelial cell ACE2 protein in inflamed tissues by immunostaining, with a progressive cytosolic translocation with increasing inflammation [72]. Furthermore, in primary human airway epithelium upon allergic inflammation, glycosylation of full-length ACE2 is reduced, thereby limiting expression on the apical side of ciliated cells exposed to viral infection [73].

Microarray transcriptomic analyses have reported reduced ACE2 expression in the small intestine of CD patients, while elevated levels have been observed in the colonic tissue of individuals with UC compared to controls [74]. Similarly, other studies have shown increased levels of ACE2 transcript in the inflamed colon of both UC and CD patients, but decreased expression in the inflamed ileum of CD patients [75]. In line with these findings, a database-mining study also reported elevated ACE2 expression in the colon of both CD and UC patients, and reduced levels in the ileum of CD patients [76]. Another study assessing colonic ACE2 expression using RNA sequencing and quantitative RT-PCR reported elevated levels in a subset of adult CD patients with a poor prognosis [77].

However, other studies analyzing mucosal biopsies from IBD patients have reported lower ACE2 expression compared to controls, as determined by RT-PCR [78]. These findings are consistent with previous reports showing reduced transcript levels of both ACE2 and TMPRSS2 in IBD patients [79]. In addition, no significant differences in ACE2 protein levels between IBD patients and controls have been reported in a quantitative proteomic study using intestinal tissue biopsy samples obtained during colonoscopy [80]. Similarly, another proteomic analysis found that ACE2 expression remained unchanged in the gut of patients with UC compared to control individuals [81].

In summary, analyses of intestinal biopsy samples have yielded inconsistent results, reflecting not only inter-study variability but also the inherently heterogeneous expression patterns of ACE2 and TMPRSS2 throughout the gastrointestinal tract and differences in cellular location. As such, assessing the levels of these proteins in the plasma could provide a more suitable means of quantification to determine susceptibility to SARS-CoV-2 infection.

## 4. Levels of ACE2 and TMPRSS2 in IBD Plasma

As mentioned, assessing the levels of ACE2 and TMPRSS2 species in the plasma of IBD patients could be potentially informative to gain a more systemic perspective on their potential vulnerability to COVID-19. Studies conducted using methodologies unable to discriminate between the different ACE2 species reported different results. As such, an early study reported higher levels of circulating ACE2 activity, estimated by an enzymatic assay, in patients with IBD compared to controls [82], whereas in a more recent study, serum sACE2 levels, estimated by ELISA, changed minimally in IBD patients [78].

In this complex scenario, we recently characterized changes in CD patients in different species of ACE2 and TMPRSS2 present in circulation [59]. In this study, only ACE2 full-length species appeared significantly decreased in the plasma of CD patients before treatment onset, as compared with age-matched controls, while levels of cleaved fragment remained unaltered. This reduction on levels of soluble full-length ACE2 may reflect decreased overall tissular expression or enhanced ACE2 shedding mediated by IBD condition. Indeed, a quotient between ACE2 fragment and ACE2 full-length species was found to discriminate between CD patients and control subjects (summarized in Figure 4B).

Moreover, there are several alternative transcripts of ACE2 encoding short isoforms, some missing parts of the cytoplasmic and collectrin domains, but also other species lacking the ectodomain SARS-CoV-2 spike protein binding sites [83]; thus, resulting in shorter forms with varying abilities to interact with the SARS-CoV-2 spike protein. Therefore, the independent estimation of these variants is relevant to determine if changes contribute to susceptibility to SARS-CoV-2 infection.

As discussed, different ACE2 species could be relevant in both a functional sense and as modulators of distinct pathologies. ACE2 can be cleaved through different means, such as the ACE2 sheddase ADAM17/TACE. This sheddase has been classically associated with inflammation, since it also processes TNFα and releases the soluble biologically active form, thus attracting interest in the IBD field, at least from a therapeutic view. A loss-of function/deletion of ADAM17 has also been associated with bowel disease [84,85]. However, alterations of ADAM17 associated with CD or UC are unclear. ADAM17 activity was demonstrated in all cell lines and in cells of controls or IBD patients, irrespective of disease activity [86], but an early study reported increased ADAM17 activity in the colonic mucosa of UC patients [87]. Further studies on ADAM17 expression in intestinal mucosa failed to demonstrate changes in IBD patients, regardless of medication [78,88]. The contribution of other members of the ADAM family to IBD has also been proposed, particularly ADAM19, the RNA transcript and protein levels of which appear upregulated in intestinal mucosa from patients with UC and, to a lesser extent, in patients with CD compared with controls [89].

As mentioned above, TMPRSS2 is also capable of cleaving ACE2, in competition with ADAM17 [51]. Therefore, elevated TMPRSS2 levels in IBD could exert a dual effect on SARS-CoV-2 susceptibility by influencing both ACE2 processing and spike protein priming. However, studies have reported decreased expression of TMPRSS2 in the intestinal mucosa of IBD patients compared to healthy controls [78,79]. In our analysis of plasma from CD patients, the active TMPRSS2 protease fragment remained unchanged, while a reduction was observed in the prodomain fragment (summarized in Figure 4B). This finding suggests a potential impairment or reduction in the zymogen-to-active protease conversion of TMPRSS2 in CD patients [59]. Therefore, if increased ACE2 shedding occurs in the context of IBD, it is likely to be independent of TMPRSS2 activity. Our data also indicates that alterations in TMPRSS2 levels are not associated with increased susceptibility to SARS-CoV-2 infection in these patients.

## 5. Effects of IBD Therapies on ACE2 Levels

Most of the studies commented above include patients with IBD that are under treatment, and the different therapies can differentially affect ACE2 or TMPRSS2 [90]. Immunosuppression, coupled with increased ACE2 and TMPRSS2, impairs effective immune responses against SARS-CoV-2 and increases the likelihood of severe immune response, such as cytokine storms [91]. Thus, initial concerns were raised regarding immune-suppressor therapies however observational studies conclude that treatment with biologic drugs does not represent a risk factor for the SARS-CoV-2 infection in patients with IBD [92,93], and a meta-analysis data indicates that susceptibility to COVID-19 did not increase with any drugs for IBD [27]. Thus, based on the currently available data, immunomodulatory and biological therapies can be continued in patients with IBD in remission suffering COVID-19 infection (discussed in [94]). Attention should be paid to new results on the influence on ACE2 and TMPRSS2 levels and proteolytic processing in the dynamic pandemic situation and development of new IBD therapy approaches [95].

Indeed, commonly used IBD therapies, both biologic and non-biologic, do not appear to significantly affect ACE2 or TMPRSS2 expression patterns in uninflamed intestinal tissue [62,78], although one study reported downregulation of colonic ACE2 expression in IBD patients who responded to anti-TNFα therapy [76]. None of these studies demonstrated an increased susceptibility to SARS-CoV-2 infection associated with changes in ACE2 or TMPRSS2 expression induced by IBD treatment. It is important to note that these studies did not evaluate plasma levels of these proteins or investigate alterations in their proteolytic processing, such as differences between full-length and cleaved fragments.

In our previous study [59], we found that CD patients treated with adalimumab or azathioprine exhibited a recovery of circulating full-length ACE2 species. Interestingly, patients treated with azathioprine also showed a decrease in one of the ACE2 fragments. However, patients receiving infliximab or ustekinumab continued to show reduced levels of full-length ACE2, along with decreases in its cleaved fragments. These alterations were modest in magnitude, suggesting that IBD therapies may modulate ACE2 proteolytic processing and contribute to the restoration of physiological regulation. Anyhow, none of the changes can be interpreted in the view of increased susceptibility to SARS-CoV-2 infection. Thus, no evidence points to the need for drug repositioning or the management of COVID-19 in patients with IBD.

Regarding TMPRSS2, none of the therapies studied resulted in significant changes in the levels of the active protease domain [59]. However, given that TMPRSS2 protease activity is driven by the fragment generated after autoproteolytic cleavage, not directly depending of zymogen/mRNA abundance, to determine enzyme activity assays appears revelant. Moreover, the regulation of how TMPRSS2 is able to autoactivate by autoproteolytic cleavage requires further investigation, also in the context of IBD. Interestingly, individuals with Down syndrome (DS) have TMPRSS2 triplication and show increased vulnerability to SARS-CoV-2 infection [96]. Associations between IBD and Down syndrome have been reported, although very sparsely [97,98], and poorer clinical outcomes may be related with immune dysregulation and a high prevalence of comorbidities.

Therapies for COVID-19 include use of human recombinant soluble ACE2 to act as decoy receptors for binding and neutralizing SARS-CoV-2 [99,100] and inhibition TMPRSS2 activity to prevent spike protein priming [101,102]. The potential impact of such therapies in infected IBD patients should be monitored.

Finally, further research focusing on the immune response to COVID-19 vaccination in biologically treated IBD patients, as well as on the long-term consequences of SARS-CoV-2 infection in this population, will be essential to better understand their vulnerability to COVID-19. Longitudinal studies on the expression and regulation of processing of ACE2 and TMPRSS2 over the course of the CD and UC are needed.

## 6. Conclusions and Final Remarks

In conclusion, individuals with IBD do not exhibit molecular changes in tissular or circulating ACE2 nor TMPRSS2 consistent with increased vulnerability to SARS-CoV-2 coronavirus cell entry in the gastrointestinal tract. Further studies addressing which species of ACE2 and TMPRSS2 could be altered in plasma from IBD patients infected by SARS-CoV2 can improve our knowledge about the role of these proteins in virus infectivity and whether this condition confers high susceptibility to SARS-CoV-2 cell entry. In this regard, it has been reported that ACE2 activity in plasma from SARS-CoV-2 positive patients is unchanged compared to SARS-CoV-2 negative patients [103]. In a cross-sectional retrospective multicenter study in COVID-19 patients with and without IBD, the levels of serum ACE2 levels were higher in IBD patients [104].

One key insight emerging from our recent studies is the critical need to evaluate not only total levels of ACE2 and TMPRSS2, but also their specific molecular forms. The distinction between ACE2 soluble species, either as proteolytic fragments or full-length forms, is essential for understanding its dual role as a viral entry receptor and a potential protective decoy in circulation. Similarly, the functional relevance of TMPRSS2 must be interpreted in light of its activation status, given that the enzymatically active protease domain appears to be selectively modulated in disease contexts. From a methodological standpoint, we advocate for future studies to employ molecular approaches that allow the discrimination of ACE2 and TMPRSS2 forms (such as quantitative Western blotting) in both tissue biopsies and peripheral fluids. Moreover, such analyses should be conducted within stratified and longitudinal patient cohorts, taking into account disease subtype, anatomical location of inflammation, treatment modality, and clinical course. Addressing this will require mechanistic and translational studies that go beyond observational data.

Finally, the potential utility of soluble ACE2 and TMPRSS2 molecular forms as surrogate biomarkers of mucosal inflammation, epithelial integrity, or viral vulnerability in IBD remains an open question. Interestingly, in our recent study [59] the estimation of a quotient between the largest ACE2 fragment and the full-length form gave the greatest discrimination between CD patients and controls. However, in plasma from COVID-19 patients [52] and in CSF of patients with COVID-19 encephalopathy [53] the shortest ACE2 fragment is the one that specifically display increases, which we also defined in an alternative quotient. Therefore, assessing the changes in ACE2 fragments could be useful for evaluating progression of IBD or of COVID-19 when IBD patients are infected.

More efforts are needed to clarify the interplay between chronic intestinal inflammation and viral pathogenesis but could also identify novel therapeutic or diagnostic avenues relevant to future pandemics and immune-mediated diseases.

## Data Availability

Not applicable.

## Abbreviations

The following abbreviations are used in this manuscript:

ACE2	angiotensin-converting enzyme 2
ADAM	a disintegrin and metalloprotease
Ang	angiotensin
CD	Crohn’s disease
CSF	cerebrospinal fluid
ELISA	enzyme-linked immunosorbent assays
IBD	Inflammatory Bowel Disease
JAK	Janus kinase
TNFα	tumor necrosis factor-α
UC	ulcerative colitis
